# Supplementary material for: Identification of ABF/AREB gene family in tomato (Solanum lycopersicum L.) and functional analysis of ABF/AREB in response to ABA and abiotic stresses
Source: PeerJ. 2023 May 4;11:e15310. doi: 10.7717/peerj.15310 (PMC10164373; doi:10.7717/peerj.15310)
Supplement: Supplemental Information 1 [file peerj-11-15310-s001.docx]

>AtABF1

MGTHIDINNLGGDTSRGNESKPLARQSSLYSLTFDELQSTLGEPGKDFGSMNMDELLKNIWTAEDTQAFMTTTSSVAAPGPSGFVPGGNGLQRQGSLTLPRTLSQKTVDEVWKYLNSKEGSNGNTGTDALERQQTLGEMTLEDFLLRAGVVKEDNTQQNENSSSGFYANNGAAGLEFGFGQPNQNSISFNGNNSSMIMNQAPGLGLKVGGTMQQQQQPHQQQLQQPHQRLPPTIFPKQANVTFAAPVNMVNRGLFETSADGPANSNMGGAGGTVTATSPGTSSAENNTWSSPVPYVFGRGRRSNTGLEKVVERRQKRMIKNRESAARSRARKQAYTLELEAEIESLKLVNQDLQKKQAEIMKTHNSEVITFFLYLSKGIFEAASIAGQKTMLEKNPYRSVVRR

>AtABF2/AtAREB1

MVQIQLLGGSRFCRKMDGSMNLGNEPPGDGGGGGGLTRQGSIYSLTFDEFQSSVGKDFGSMNMDELLKNIWSAEETQAMASGVVPVLGGGQEGLQLQRQGSLTLPRTLSQKTVDQVWKDLSKVGSSGVGGSNLSQVAQAQSQSQSQRQQTLGEVTLEEFLVRAGVVREEAQVAARAQIAENNKGGYFGNDANTGFSVEFQQPSPRVVAAGVMGNLGAETANSLQVQGSSLPLNVNGARTTYQQSQQQQPIMPKQPGFGYGTQMGQLNSPGIRGGGLVGLGDQSLTNNVGFVQGASAAIPGALGVGAVSPVTPLSSEGIGKSNGDSSSLSPSPYMFNGGVRGRKSGTVEKVVERRQRRMIKNRESAARSRARKQAYTVELEAEVAKLKEENDELQRKQARIMEMQKNQETEMRNLLQGGPKKKLRRTESGPW

>AtABF3

MGSRLNFKSFVDGVSEQQPTVGTSLPLTRQNSVFSLTFDEFQNSWGGGIGKDFGSMNMDELLKNIWTAEESHSMMGNNTSYTNISNGNSGNTVINGGGNNIGGLAVGVGGESGGFFTGGSLQRQGSLTLPRTISQKRVDDVWKELMKEDDIGNGVVNGGTSGIPQRQQTLGEMTLEEFLVRAGVVREEPQPVESVTNFNGGFYGFGSNGGLGTASNGFVANQPQDLSGNGVAVRQDLLTAQTQPLQMQQPQMVQQPQMVQQPQQLIQTQERPFPKQTTIAFSNTVDVVNRSQPATQCQEVKPSILGIHNHPMNNNLLQAVDFKTGVTVAAVSPGSQMSPDLTPKSALDASLSPVPYMFGRVRKTGAVLEKVIERRQKRMIKNRESAARSRARKQAYTMELEAEIAQLKELNEELQKKQVEIMEKQKNQLLEPLRQPWGMGCKRQCLRRTLTGPW

>AtABF4/AtAREB2

MGTHINFNNLGGGGHPGGEGSSNQMKPTGSVMPLARQSSVYSLTFDELQNTLGGPGKDFGSMNMDELLKSIWTAEEAQAMAMTSAPAATAVAQPGAGIPPPGGNLQRQGSLTLPRTISQKTVDEVWKCLITKDGNMEGSSGGGGESNVPPGRQQTLGEMTLEEFLFRAGVVREDNCVQQMGQVNGNNNNGFYGNSTAAGGLGFGFGQPNQNSITFNGTNDSMILNQPPGLGLKMGGTMQQQQQQQQLLQQQQQQMQQLNQPHPQQRLPQTIFPKQANVAFSAPVNITNKGFAGAANNSINNNNGLASYGGTGVTVAATSPGTSSAENNSLSPVPYVLNRGRRSNTGLEKVIERRQRRMIKNRESAARSRARKQAYTLELEAEIEKLKKTNQELQKKQAEMVEMQKNEQLKETSKRPWGSKRQCLRRTLTGPW

>AtABI5/AtDPBF1

MVTRETKLTSEREVESSMAQARHNGGGGGENHPFTSLGRQSSIYSLTLDEFQHALCENGKNFGSMNMDEFLVSIWNAEENNNNQQQAAAAAGSHSVPANHNGFNNNNNNGGEGGVGVFSGGSRGNEDANNKRGIANESSLPRQGSLTLPAPLCRKTVDEVWSEIHRGGGSGNGGDSNGRSSSSNGQNNAQNGGETAARQPTFGEMTLEDFLVKAGVVREHPTNPKPNPNPNQNQNPSSVIPAAAQQQLYGVFQGTGDPSFPGQAMGVGDPSGYAKRTGGGGYQQAPPVQAGVCYGGGVGFGAGGQQMGMVGPLSPVSSDGLGHGQVDNIGGQYGVDMGGLRGRKRVVDGPVEKVVERRQRRMIKNRESAARSRARKQAYTVELEAELNQLKEENAQLKHALAELERKRKQQVKTPIEFALLRWLQYFGQKKTTKWNCWFMVAVF

>AtDPBF2

MSVFESETSNFHVYNNHEIQTQPQMQTFLSEEEPVGRQNSILSLTLDEIQMKSGKSFGAMNMDEFLANLWTTVEENDNEGGGAHNDGEKPAVLPRQGSLSLPVPLCKKTVDEVWLEIQNGVQQHPPSSNSGQNSAENIRRQQTLGEITLEDFLVKAGVVQEPLKTTMRMSSSDFGYNPEFGVGLHCQNQNNYGDNRSVYSENRPFYSVLGESSSCMTGNGRSNQYLTGLDAFRIKKRIIDGPPEILMERRQRRMIKNRESAARSRARRQAYTVELELELNNLTEENTKLKEIVEENEKKR

RQEIISRSKQVTKEKSGDKLRKIRRMASAGW

>AtDPBF3/AtAREB3

MDSQRGIVEQAKSQSLNRQSSLYSLTLDEVQNHLGSSGKALGSMNLDELLKSVCSVEANQPSSMAVNGGAAAQEGLSRQGSLTLPRDLSKKTVDEVWKDIQQNKNGGSAHERRDKQPTLGEMTLEDLLLKAGVVTETIPGSNHDGPVGGGSAGSGAGLGQNITQVGPWIQYHQLPSMPQPQAFMPYPVSDMQAMVSQSSLMGGLSDTQTPGRKRVASGEVVEKTVERRQKRMIKNRESAARSRARKQAYTHELEIKVSRLEEENERLRKQKEVEKILPSVPPPDPKRQLRRTSSAPF

>AtDPBF4/AtEEL

MSSQLKATYMGSIRGNIEEPISQSLTRQNSLYSLKLHEVQTHLGSSGKPLGSMNLDELLKTVLPPAEEGLVRQGSLTLPRDLSKKTVDEVWRDIQQDKNGNGTSTTTTHKQPTLGEITLEDLLLRAGVVTETVVPQENVVNIASNGQWVEYHHQPQQQQGFMTYPVCEMQDMVMMGGLSDTPQAPGRKRVAGEIVEKTVERRQKRMIKNRESAARSRARKQAYTHELEIKVSRLEEENEKLRRLKEVEKILPSEPPPDPKWKLRRTNSASL

>AtbZIP15

MDSYWRLKNLVNDLPVSTSLSRQGSIYSWTVDQFQTSLGLDCGSMNMDELVKHISSAEETQEGSQRQGSTTLPPTLSKQNVGEVWKSITEEKHTNNNGGVTNITHLQGQQTLGEITLEEFFIRAGARGGNTNGGSIHDSSSSISGNPHTSLGVQIQPKAMVSDFMNNMVPRSHDSYLHQNVNGSMSTYQPQQSIMSMPNGYSYGKQIRFSNGSLGSGNQSLQDTKRSLVPSVATIPSEAITCSPVTPFPTLNGKQKINGESSLLSPSPYISNGSTSTRGGKINSEITAEKQFVDKKLRRKIKNRESAARSRARKQAQTMEVEVELENLKKDYEELLKQHVELRKRQMEPGMISLHERPERKLRRTKSDIK

>PdABF1

MGTNMNFKGFGNDPPGINPLARQSSIYSLTFEELQNTIGGSGKDFGSMNMDELLKSIWTA

EETQIMAPSGGGAGGQNGLGLGGGSLQRQGSLTLPRTLSQKTVDEVWKNISKEGTGPGAS

NMPQRQQTLGEMTLEEFLVKAGVVREEAQLAPKPANNGAGFFGDLSRFGNTGGNLDFEFQQTNRGVGVMGNRETNSQVPNQASNLPLNANGVRSNQQQQQLPQQQQIFPKQQPVTYNTSPLPMGPNAQLGSPGMRGGIMGIGDQGLNGTLVQSSGMGMVGLGAAGAVRVATGSPANQLSSDGIGKSNGTDTSSVSPVPYVFNGGFRGRKGGGPVEKVVERRQRRMIKNRESAARSRARKQAYTMELEAEVAKLKEENQELQKKQAEMMEMQKNQDMEIINLQRGKKRCLRRTQTGPW*

>PdABF2

MEDRTLESGNGGERPQFPPLARQESYNLSNLDEAQSHLGNINSKPLNGMHFDALLKNVIS

VEEGQQLQNPSSSSLPASFFLGNFNLNGALSRKTADEVWKEIAHHEHVNTVVANESLQQR

LSTIGETPATPEHFLVRAGVINIGNQPSLMNAAQPIMGIDPTVVSQQTDWLQFQMAAVQQ

QMTMLDSNLKVRESVYENSAVNFDYSENQVGMSMPMPAISASSCESRATAVRKRHFSDEMKERTIERRQKRMIKNRESAARSRARKQAYTNELEHEVFQLGKVNSWLKKQKEVEMILASNPTSMPKYQLRRTSSAPY*

>PdABF3

MGIQTMGSQGGADGNCKQSQFQPLARQNSIYSLTLDEVQNQLGDLGKPLSSMNLDELLKNVWSAEANQTMGMDIEGTTLVNQATLQRQASLSLTSALSKKTVDEVWRDIQQSKNNEEKKSQERQRTLGEMTLEDFLVKAGVVAEAEASSDKKCSAPLAVVDANVGSQFPQGQWLQYQQPQYQHPQQSMMGVYMPSQPIPPPLHIGAGAIMEVPYPDNQVALPSPLMGTLSDTQTPGRKRGNPEDIVEKTVERRQKRMIKNRESAARSRARKQAYTNELENKVSRLEEENERLRKQKELEKVLPSAPPPEPKYQLRRTTSAPF*

>PdABF4

MVASDESGSIGYDNDNFDFEFEPEQPLEPQEESSQDDSHHSMGNNTKQNSIFSLTLDEIQ

CKSGRNFGSMNMDEFLANIWSVEEENQTQQQPSQCDQDASDKDTTINPITLSRQGSFSIP

TPLCKKTVDEVWSEIDRSRPQHHDPDGNISNSVAPQRQQTIGEITLEDFLVKAGVVQESP

SKSKPPPPKMESTLIQQQCGETNMGNEISTCLDSSFGIGRQLFGSGFFNLQNDVPSNLSG

NGYAIGATYPMLGRQSKVIVGEPSCVSAIEKCHSLPESSSGGGAKNKKRIIDGPPEVVVE

RRQRRMIKNRESAARSRARKQAYTVELEAELNQLKEENTKLKQNVAESEQKRKQELLGKKQSTRAQKLAEKLRTMRRAVSLAW*

>PdABF5

MGFQTMASQANGQQSHFQPSPLLRQPSWYSLTLDEVKNQLGDTGKPLGSMNLDELLQNLWTAEANQSIEMDIENTSSASSLQRQASLTLARALSGKTVDEVWREIQQGQKKRYGEDMKCQDTEITLGETTLEDFLVQAGLFAEASLSPAIALDTIEVAIPQSYPHNLGLSSSPSFGTHSDTTTPGRKRDASDEYEKTVERRLRRKIKNRESAARSRARKQAYHNELASKVSRLEEENIKLKKEKEFEKMLPSALSAEPKYQLRRISSHHQMAGRPCH*

>PdABF6

MGVSESEIISHDEVESPLQSDQQATNHLFTSLGRQSSIYSLTLDEFQHTLCENGKNFGSMNMDEFLTSIWTAEENQAINSNHTNINNNHNHHNHHNSNINNIDAHMPLAEASEEKAAAIAKQPSLPRQGSLTLPGPLCRKTVDEVWSEIHKGKQAKQQNSHSSNDGVQNSEFAPRQPTFGEMTLEDFLVKAGVVREPDSMLAAGAVLPPQPQQQQQQYGMYQNSNQAVGPSFANRPVMGMGAAGAAGPSTSAAAGMPNYQGMPQNGATVVAESSGYAANGKRNGAYPAVPPPQAVCFGGRVVNGGGGYAAGQPIGMAAPVSPVSSDGMCTSQVENSGGQFGLDMGGLRGRKRILDGPVEKVVERRQRRMIKNRESAARSRARKQAYTVELEAELNQLREDNAHLKQALAELERKRKQQYFDEMQTRVQIALYDMQDREMIIEEQNWHLPPRGLKLCKLTPMLKWLQGGAND*

>PdABF7

MGSNFNFKNFGDAPPGEGNGGKAAGNFTLARQPSVYSLTFDEFQNTIGGLGKDFGSMNMDELLKNIWTAEETQGVTSTSGAGEGSAPGGNLQRQGSLTLPRTLSQKTVDEVWKDLIRETSDAKYNTVAMGSNLPQRQQTLGEMTLEEFLVRAGVVREDVQPVVRPNNSGFYGELYRPNNHNGLAPGFQQPSRTNGLLGNRVADNNNSVLNQSPNLALNVGGVRSSQQQTQQLPPQQQPLFPKPTNVAFAPSMHLTNNAQLTSPRTRGPMTGVVEPSMNTVFTQVGGFPGAGIGMTGLGTGGGAVAARSPTNQISPDVIAKSSGDTSSLSPVPYMFSRGRKSNGALEKVVERRQRRMIKNRESAARSRARKQAYTLELEAEVAKLKEMNEELQRKQTEIMEMQKDQILETVKRQRGGKRQCLRRTLTGPW*

>StAREB1

MGSYMNFKNVADTPQLESNGGKSIGNGDFPLARQSSIYSLTFDELQTTFSGLGKDFGSINMEELLKSIWTAEESQAVTSSTGGGGDGNAPVGNLQRQGSLTLPRTLSQKTVDEVWRNFQKETTVCTKDGSDTGKSNFGQRQSTLGEMTLEEFLVKAGVVGEDMQSTSNSSGITFNNGSSQQNNNNGFNIAFQQPTQNTGLLINQIAANNMLNVVDPTASQQQQPQKQQPLFPKQTTVAFSSPMQLSNNGHLASPRTRAPAVGMSSPSVNANMAQGGVMGMTGFYNGVSPAKGGSPGNDFVARSNVDTSSLSPSPYAYSEGGRGRRSGSSLEKVVERRRRRMIKNRESAARSRARKQAYTLELEAEVAKLKKINEELRKKQAEIIENQKNQLTDKRNMPRGYKLRCLRRTLTGPW

>StAREB2

MGSNYHFKNFGNEPPGEGGSGGGGDGGKQLGNFGLPRQSSIYSLTFDEFLSSTGGSGKDFGSMNMDELLKNIWNAEENQTIGGPGINGQEVGVPRGHLQRQGSLTLPRTLSHKTVDEVWRDMSKEHGGGKDGNSVGVPPNIPQTQRQQTFGEITLEEFLVRAGVVREDAQFAAKSNNTGGIFGDLSYAGNNTGLAFGYQQANNRNTGLMAGSIPNKNGETVIQTANLPLNVNGVRSTQQQLRPQQLQQNHQPQQQQPQQQPIFPKQPALPYGAPMAIPNSGQLGSPGMRVGMVGIPDPALNSNFIQGNALMGGGMNMVGLGASGVTVATASPGVSSSDGLGKSNGDTPSVSPVPYVFNGGLRGRKYSTVEKVVERRQRRMIKNRESAARSRARKQAYTMELEAEVAKLKEENDELQKKQEEMLEMQKNQVMEMMNLHKGAKRRCLRRTQTGPW

>StAREB3

MGSYLNFKNFTDTPQIESNGEKSLGSGSFPLARQSSMYSLTFEELQSTCGLGKDVGSMNLEDLLRNIWTAEDSQALASSAGVGEGRMAAGNLQRQGSLTLPQTLNQRTVDEVWRDFQNETTVSSNLGQKQSNLGEMTLEEFLVRAGVVRDDNQPNGTSNNAGFAGVLGEPSSNNNALNIAFQQPTQWPGLLSNQFAESNMLNVVSVKSSQQQPHQQQPLIPKQRNVDFASPMQLGNNCQLASPRVRALVVSTSSRSVNTTVVQGGVMQNGVTGMAGLRSGVTFAPAKGGSPGNQLSSEMISNDKLNRPSPSPSPYAFNEGRRGRKSCSSLEKVVERRHKRMIKNRESAARSRARKQAYTLELEAEVAKLKEINEELQEKQAEFIDPQKNQLLEKMNMPLGSKLRCLRRTLTGPW

>StAREB4

MGSYLNFKNFADTSQPESSGNNSNFSLARQSSIYSFTFDELQSTCGLGKDFGSMNMDDLLKNIEESQAFPSSAAAGGNLQRQGSLTLPRTLSQRTVDEVWKDFQKESVIANDVSGTGGSNFGQRESTLGEMTLEEFLVRAGAVREDMQPAGYSNDVTFASGFTQPSSSVTIAFQQATQNPGHQIAGNNIFNVVSTTTSSTQQPLFPKQTTVEFASPLQLGGSPGTRPPMSNPSANTSSVMQGGVMTMPVKGVSPGNIDTSSLSPSPYACGEGGRGRRSCTSFEKVVERRRKRMIKNRESAARSRDRKQAYTLELEAEVAKLKEIKQELQKKQAEFIEKQKKQLLEKMNVPWENKLICLRRTVTGPW

>StABI5

MGVPESEMVSQSEVQSPLQPDQNQNKNNPFPSLGRQASIYSLTLDEFQHTVCESGKNFGSMNMDEFLNSIWTAEENQAHAHAHVHAQPHCQAASTGEATSAPRFALGQGNVSLQKAIVEQPSLPRQGSLTLPAPLCSKTVDEVWSEIHKTQQEQQQNNGCSIQNTGNGSSTQRQTTFGEMTLEDFLVKAGVVREQGNSAPAPPQQQSYMMYPNSANPTMARPVIGLGGVTGGVGVGVAIPGYPPLPQTGVVEAPVYPMSMKRGSGFPQQPTPVYGGRMGNGSGVGYGQVVQSVTGMGSPLSPVSSDGLCVNQIDNVGQYGLEIGMRGGRKRVLDGPVEKVVERRQRRMIKNRESAARSRARKQAYTVELEAELNQLKEENAHLKQALAELERKRKQQYFDEAKTKAQTKAQKANGKLRGMRRSFSCP

>StABL1

MGSQGGGGGGGGGVGVNSIGATQTQAQAQAHAQDPKTNALARQGSLYSLTLDEVQNQLGDLGKPLSSMNLDELLKTVWTVEASQGMGGTDYGVLQHGQDASGSSLNRQSSITLTSDLSKKTVDQVWQDIQQGHKRDSIDRKAQERQPTLGEMTLEDFLVKAGVVAESTPGKKSSGSVLGVDSMALPQQQAQWSQYQMHAMHQLPPQQQQQNMLPVFMPGHSVQQPLTIVSNPTIDAAYPESQMTMSPTALLGTLSDTQTLGRKRVAPDDVVEKTVERRQKRMIKNRESAARSRARKQAYTHELENKVSRLEEENERLKRQKEIEQVLPSVPLPEPKYQLRRTSSAPV

>StABL2

MVIQGMGSQGGDMSRDLSKKTVDEVWQDIQQGVKTDNVDKRSQERQLTLGEITLEDFLVKAGVIAESTQGKRISGLVFGVDSMSLTQQAQWPHYQIPAMQQVPEQQHQQQQQNIPPVFMPGHPIQQPLPVVANPIMDATYPETQVTMSPAHIMGTLSDTQTSGRKRVAPHDVAENSVERRQKRMIKNRESAARSRARKQAYTHELENKVSFLEEENEKLKRQKEMEDILPSVPPPEPKYQLRRTSSGPI

>SlABF1

MNSQERELTLGETTLEDYLVKAGLFVADASLGHTMSLDNPTAMQNFVPPIGLSPSPSLSDTPVSDRKRGAMDIDKTIDRRLRRKIKNRESAARSRARKQAYHNELVNKVSHLEEENMKLKKEKELENMLSELSSEPRYQLRRTTSF

>SlABF2

MASKVMPSASTTPNSDRSLHPQNPSSSLNHSHPSRNFDSMNMDEILKNIYSDSDPFACSVSATAAVHTPSATAAGVGDVGPTKTVDEVWREIVAGGGGGGGSREPEMTLEDFLTKAGAVTEEDVRVPVIAPPPPPPPPPATGAPSARGFVVDNMMGTGNCQFPVAMQNGPGGYGMEPQPHMGFGNGVVAITGSGSGSGRGKRRSTVEELPADRATQQKQRRMIKNRESAARSRERKQAYTVELESLVTQLEEENARLLREEEEKNKERLKQIMENLIPVVEKRRPPRVLRRVRSMSW*

>SlABF3

MGSYMNFKNITDKPQAESNGGKSVGNGDIPLARQSSIYSLTFDELQTTFSGLGKDFGSINMEELLKSIWTAEESQAATSSTGGGEDGIAPVGNLQRQGSLTLPRTLSQKTVDEVWRNFQKETTVCTPDGSETGKSNFGQRQSTLGEMTLEEFLVKAGVVREDMQSTSNSSGITFNNGLSQQNNNNGFNIAFQQPTQNNGLLINQIAANNMLNVVGATASQQQQPQQQQPLFPKQTTVAFASPMQLSNNGHLASPRTRAPAVGMSSPSVNASMAQGGVMGKTGFHNGVSPAKVGSPGNDFI

ARSNVDTSSLSPSPYAFSEGGRGRRSGSSLEKVVERRRRRMIKNRESAARSRARKQAYTLELEAEVAKLKEINEELRKKQAEIIEKQKNQLTDKRNMTCGYKLRCLRRTLTGPW

>SlABF4

MASTKLMASSASRNSDRRKSSAASSSSSSTMPNDLLNQQYSNNGSNNSSNLEATSMTVDGFLRNVYGEGQGTEANTLLNANITLLDAAGAITPISDSETATVSGIPLVRRTVDDVWREIVEGKREQRRAAVAGCKEEAVDEIMTLEDFLVKAGAVEEEALAGEGPVQGEVKVELGTERLSGGIFAFDSPYMAMPQQSVQGYGNGIDVIGGGRGKRKAILEPLDKAALQRQRRMIKNRESAARSRERKQAYQVELESIAVRLEEENEQLLKEKEERTRAHYKQIIEKVIPVVEKRKPPRVLRRVCSMQW*

>SlABF5

MGSNYHFKNFGNEPPGEGGSGGGGKQPGNFGLPRQPSIYSLTFDEFLSSTGGSGKDFGSMNMDELLKNIWNAEENQTIGGPGINGQEVGVPGGHLQRQGSLTLPRTLSHKTVDEVWRDMSKEHGGGKDGNSVGVPPNIPQTQRQQNLGEITLEEFLVRAGVVREDAQFAAKSNNAGGIFGDLSYAGNNTGLAFGYHQANSRNTGLMAGSIPNKNGETVIQSANLPLNVNGVRSTQQQLRPQQLQQNQQSQPQQQPIFPKQPALPYGAPMAIPNSGQLGSPGMRAGMVGIPDPALNSNFIQGASLMGGGMNMVGLGASGVTVATASPGVSSSDGLGKSNGDTPSVSPVPYVFNGGLRGRKYSTVEKVVERRQRRMIKNRESAARSRARKQAYTMELEAEVAKLKEENDELQKKQEEMLEMQKNQVIEMMNLQKGAKRRCLRRTQTGPW*

>SlABF6

MGVPESEMVSQSEVQSPLQQDQNQHKNNPFPSLGRQASIYSLTLDEFQHTVCESGKNFGSMNMDEFLNSIWTAEENQAHAHAQPHCQAASTGEATSAPRFALGQGNVSLEKAIVEQPSLPRQGSLTLPAPLCSKTVDEVWSEIHKTQQEQQQNNGCNIQNTGNGSSTQRQATFGEMTLEDFLVKAGVVREQGNSAPAPPQQQSYMMYPNSANPTMAAMARPVIGLGGVTGGVGVGVSIPGYPPLPQTGVVEAPVYPMSMKRGSGFPQQSTPVYGGRMGNGSGVGYGQVVQGVAGMGSPLSPVSSDALCVNQIDSGGQYGLEIGMRGGRKRVLDGPVEKVVERRQRRMIKNRESAARSRARKQAYTVELEAELNQLKEENAHLKQALAELERKRKQQYFDEAKMKAQTKAQKANGKLRGMRRSLSCP*

>SlABF7

MGSGSFSLTRQSSMYSLTFEELQSTCGFRKDVGSMNLEDLLKNISTAEESRGLASSAGVGEGSMAAGTLQRQGSLTLPRTLNQRTVDEVWRDFQNETTVSSNLGQKQSNLGEMTLEEFLVRAGVVRDDNQPNVTSNNVGFTGVLGEPSSNNNALNIAFQQPTQSPGLLSKFSESNMLNVVNVKSSQQQPQQQQPLIPKKRNVDFASTMQLGNNCQLASPRARAVVVSTSSRSVHATVVQGGVMQNGVKGMAGLLSGVTFAPGGSPGNQLSSEMISKDKLYRSFLSPSPYAFNEGGRGRKSCSTLEKVVERRHKRMIKNRESAARSRARKQAYTFALEAEVAKLKEINEKLQKKQAEFIDSQKNQVMASFTTNSKYLSL*

>SlABF8

MVIQEMGSRGGGEAKANALATQGSLYSLTLDEVRNQLGNCGKPLNSMNLDEFVKTVWTIESNQEVVGGNDYGPVQQGASQHHPSSITMSRDLSKKTVDEVWQDIQQGVKIDNVDKRSQERQLTLGEITLEDFLVKAGVIAESTQGKRISGLVFGVDSMSLTQQAQWTHYQIPAMQQVPEQQHQQQQQNIPPVFMPGHPIQQPLPVVANPIMDATYPETQVTMSPAHIIGTLSDTQTSGRKRVAPRDVAENSIERRQKRMIKNRESAARSRARKQAYTHELENKVSFLEEENERLKRQKEIEDILPSVPPPEPKYQLRRTSSGPI*

>SlABF9

MGSQGGGGGGGGGVGVNSIGVTQAQAQAQAHAQDPKTNALARQGSLYSLTLDEVQNQLGDLGKPLSNMNLDELLKTVWTVEASQGMGGTDYGVLQHGQDASGSSLNRQSSITLTSDLSKKTVDQVWQDIQQGHKRDRIDRKAQERQPTLGEMTLEDFLVKAGVVAESTPGKKSLGSVLGVDSMALPQQQAQWSQYQMQAMHPLPPQQHQQQQQNMLPVFMPSHSAQQPLTIVSNPTIDAAYPESQMTMSPTALLGTLSDTQTLGRKRVAPDDVVEKTVERRQKRMIKNRESAARSRARKQAYTHELENKVSRLEEENERLKRQKEIEQVLPSVPLPEPKYQLRRTSSAPF*

>SlABF10

MGSYLNFKNFADTSQPESSGNNSNSFLAQQSSIYSFTFDELQNTCGLGKDFGSMNMDDLLKNIEESQALSSSAALGGNLQRQGSLTLPRTLSQKTVDEVWRDFQKESVVANDASGTGGSNFGQRESTLGEMTLEEFLVRAGAVQEDMQPAGYSNDVTFASGFTQPSCSVTIAFQQATQNPGHQIAANNIFNVVSTTTSSPQQPLFPKQTTVEFASPMQLGSPGKRLPMSNPSANTSSVMQGGVMTMPVKGVSPGNLDTSSLSPSPYACGEGGRGRRSCTSFEKVVERRRKRMIKNRESAARSRDRKQAYTLELEAEVAKLKEIKQELQKKQAEFIEKQKNQLLEKMNVPWENKLICLRRTVTGPW*
