# Supplementary material for: Identification of ABF/AREB gene family in tomato (Solanum lycopersicum L.) and functional analysis of ABF/AREB in response to ABA and abiotic stresses
Source: PeerJ. 2023 May 4;11:e15310. doi: 10.7717/peerj.15310 (PMC10164373; doi:10.7717/peerj.15310)
Supplement: Supplemental Information 4 [file peerj-11-15310-s004.docx]

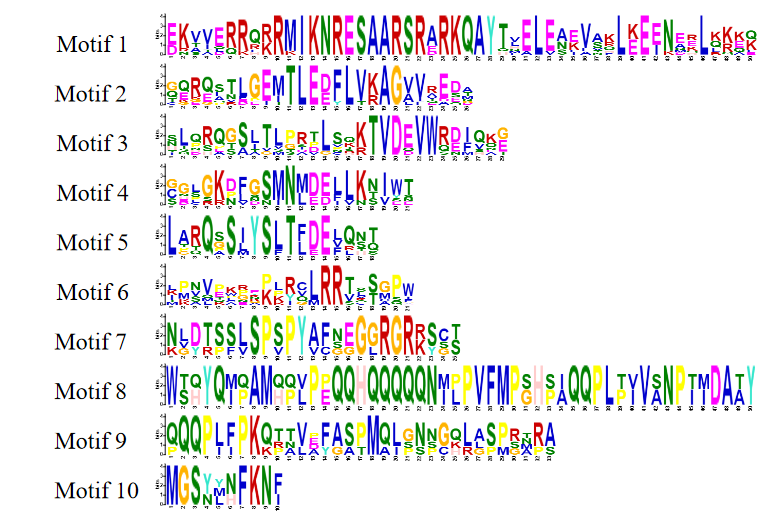


**Amino acid sequences of different conserved motifs displayed by stacks of letters at each position.**
