# Supplementary figures and images for: Identification of ABF/AREB gene family in tomato (Solanum lycopersicum L.) and functional analysis of ABF/AREB in response to ABA and abiotic stresses
Source: PeerJ. 2023 May 4;11:e15310. doi: 10.7717/peerj.15310 (PMC10164373; doi:10.7717/peerj.15310)

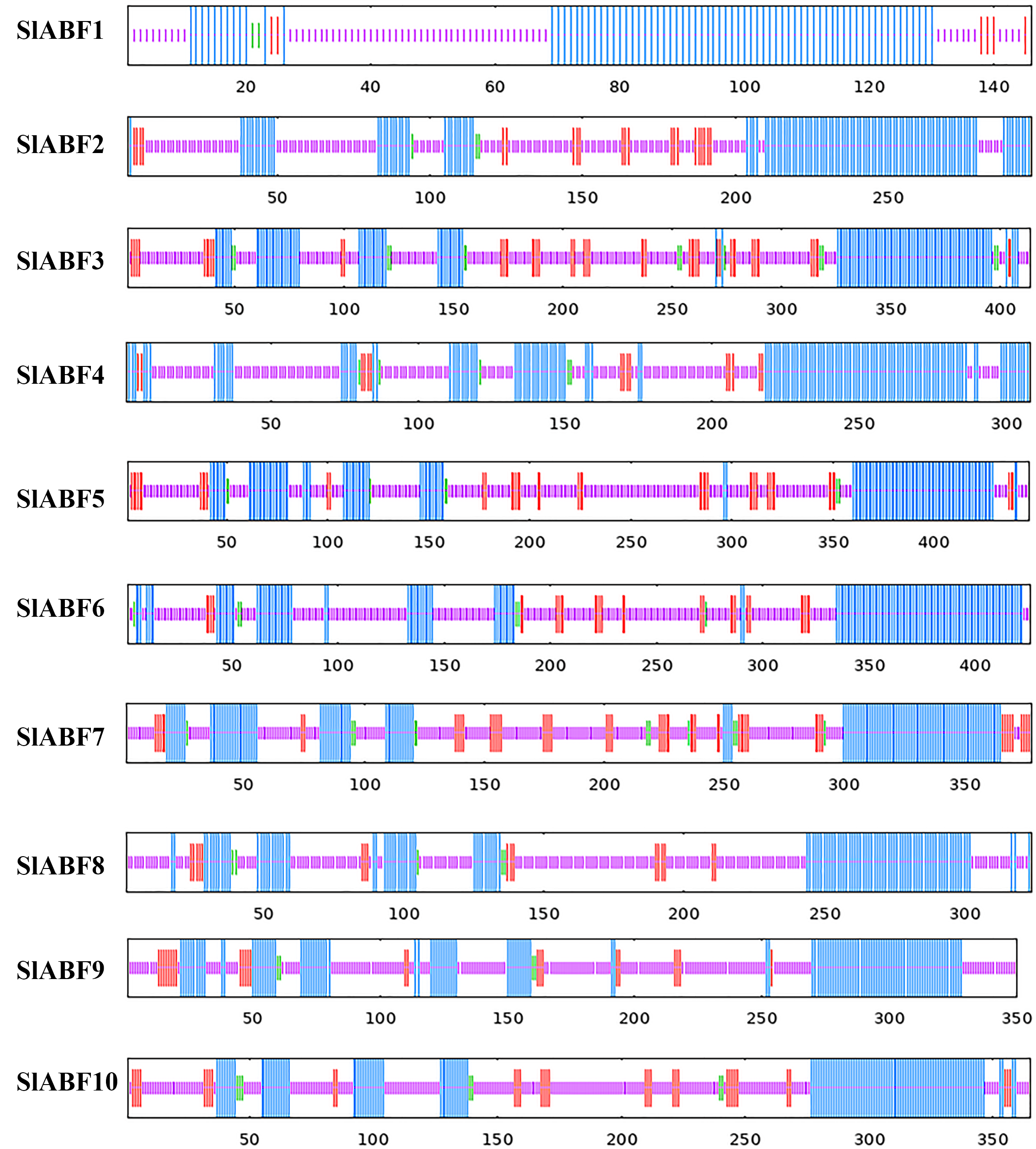

Supplement: Supplemental Information 5 [file peerj-11-15310-s005.png]
